# Microsurgical resection of giant T11/T12 conus cauda equina schwannoma

**DOI:** 10.17305/bjbms.2020.5153

**Published:** 2021-08

**Authors:** Alisa Arnautovic, Mirza Pojskic, Kenan I. Arnautovic

**Affiliations:** 1 George Washington University School of Medicine, Washington, DC, United States; 2 Department of Neurosurgery, University of Marburg, Marburg, Germany; 3 Medicinski fakultet Osijek, Sveučilište Josip Juraj Strossmayer, Osijek, Croatia; 4 Semmes Murphey Neurologic & Spine Institute, Memphis, TN, United States; 5 Department of Neurosurgery, University of Tennessee Health Science Center, Memphis, TN, United States

**Keywords:** Schwannoma, conus, cauda equina, microsurgery, gross total resection

## Abstract

In this video, we highlight the anatomy involved with microsurgical resection of a giant T11/T12 conus cauda equina schwannoma. Spinal schwannoma remains the third most common intradural spinal tumor. Tumors undergoing gross total resection usually do not recur. To our knowledge, this is the first video case report of giant cauda equina schwannoma resection. A 55-year-old female presented with paraparesis and urinary retention. Lumbar spine MRI revealed a contrast-enhancing intradural extramedullary tumor at the T11/T12 level. Surgery was performed in the prone position with intraoperative neurophysiology monitoring (somatosensory and motor evoked potentials—SSEPs and MEPs). T11/T12 laminectomies were performed. After opening the dura and arachnoid, the tumor was found covered with cauda equina nerve roots. We delineated the inferior pole of the tumor, followed by opening of the capsule and debulking the tumor. Subsequently, the cranial pole was dissected from the corresponding cauda equina nerve roots. Finally, the tumor nerve origin was identified and divided after nerve stimulation confirmed the tumor arose from a sensory nerve root. The tumor was removed; histological analysis revealed a schwannoma (WHO Grade I). Postoperative MRI revealed complete resection. The patient fully recovered her neurological function. This case highlights the importance of careful microsurgical technique and gross total resection of the tumor in the view of favorable postoperative neurological recovery of the patient. Intraoperative use of ultrasound is helpful to delineate preoperatively tumor extension and confirm postoperative tumor resection.

## VIDEO LINK

https://www.bjbms.org/ojs/index.php/bjbms/article/view/5153

Video 1

## VIDEO ANNOTATIONS

00:14— Clinical presentation and description of operative set-up

01:57—Opening of surgical site,

03:30— Opening of tumor capsule, tumor debulking, and tumor release

06:57— Identification of nerve root of origin, release of root from tumor, final gross total resection, and closure

08:45—Postoperative imaging and patient outcome

## INTRODUCTION

In this video, we highlight the anatomy involved with microsurgical resection of a giant T11/T12 conus cauda equina schwannoma. Spinal schwannoma remains the third-most common intradural spinal tumor [1,2]. Tumors undergoing gross total resection usually do not recur [1-7]. To our knowledge, this is the first video case report of giant cauda equina schwannoma resection.

## VIDEO TRANSCRIPT

This video demonstrates a microsurgical resection of a giant T11–T12 conus cauda equina schwannoma. Our patient is a 53-year-old female with paraparesis and motor strength 3 out of 5, inability to walk, urinary retention, patchy hyposthesia and lower extremities and severe back and bilateral leg pain. MRI of the lumbar spine revealed a large uniformly enhancing mass at the T11–T12 level. Differential diagnoses included schwannoma, meningioma, ependymoma, myxopapillary ependymoma, and metastasis.

The patient opted for surgery because with a large tumor in the spinal canal. Total resection removes the space-occupying lesion, prevents further tumor growth, and prevents worsening of an already severe neurological deficit. The patient was brought to the OR and placed under general endotracheal anesthesia and intubated. She was turned into the prone position. Necessary equipment included microsurgical instruments, neural monitoring, C-arm, ultrasonic aspirator, high-speed drill, dural sutures, and liga clips for the arachnoid. Intraoperative neurophysiological monitoring was performed continuously. The posterior thoracic area was prepped and draped, and C-arm fluoroscopy confirmed T10 to T12 extension of the incision. C-arm in an AP projection was used for the determination of the level, using the ribs as orientation as the patient was positioned on a carbon radiolucent table. Laminectomy of T11 and T12 was performed using a diamond drill and Kerrison rongeur.

The microscope was brought in. Gelfoam powder was used to achieve hemostasis in the epidural space. The dura was opened by a surgical knife and with Potts scissors and tacked up to the surrounding soft tissue with 4-0 Nurolon stitches (Ethicon Inc., Somerville, NJ, USA).

Initial exposure shows that the tumor is covered by some of the cauda equina nerve roots. The arachnoid is opened with a nerve hook and scissors in order to allow the mobilization of these nerve roots. Liga clips were applied to hold the arachnoid membrane to the dura. The tumor was covered with spinal nerves and was exposed initially in the left caudal part of the surgical field. The exposed part of the inferior tumor capsule has been coagulated and cotton paddies are placed around the circumference of the tumor for protection.

The first step in the microsurgical resection of the tumor is coagulation and opening of the tumor capsule with a bipolar and #15 blade. The tough tumor capsule is sharply incised to allow internal decompression. The initial debulking of the tumor has been performed using microscissors, bipolar, and tumor forceps to send enough tissue for histological analysis, including a frozen section. Blunt and sharp dissection with gentle traction and counter-traction facilitates the development of the dissection plane. With microscissors, we provided samples for the frozen section. The preliminary pathohistological diagnosis confirms the diagnosis of the schwannoma.

The caudal pole of the tumor can now be clearly seen. We started the dissection from the lower part as it was free from the cauda equina roots. Using bipolar and microscissors, the tumor is released on its inferior margin from the surrounding nerves of the cauda equina. Bipolar coagulation shrinks and devascularizes the tumor. Yasargil-controlled suction was used. Following this, further piecemeal resection of the solid tumor parts is performed. Stepwise resection of the solid parts of the tumor, along with the capsule, allows for more secure internal decompression. Debulking of the tumor is continued using an ultrasonic aspirator. This allows for greater and safer immobilization of the tumor in the surgical field. The schwannoma is now released at its cranial pole using bipolar coagulation and microscissors.

Once mobilized, further dissection and debulking with a microblade, microscissors, and an ultrasonic aspirator are performed. Debulking of the tumor and its internal decompression allows for mobilization of the tumor and identification of the tumor nerve of origin, as well as corresponding nerves. Each segmental dorsal and ventral root share a common arachnoid sheet. Dissection reveals the ventral and dorsal nerve root. In this case, the left dorsal T12 nerve root is the tumor origin, so the left T12 ventral nerve root is referred to as the corresponding root. The ventral corresponding nerve root is intact and can be preserved by careful and meticulous dissection.

The tumor single sensory nerve root of origin is not functional and is incorporated into the tumor. It is usually not possible and not necessary to preserve it. After dissection of the corresponding root off of the tumor, the tumor nerve origin has been coagulated and divided. Direct nerve stimulation previously confirmed the non-functional nature of the tumor nerve origin. Gross total resection of the tumor has been successfully performed. The dura was closed with 4-0 Nurolon running stitches. An application of previously harvested abdominal fat graft was used to prevent CSF leak and/or pseudomeningocele formation. Intraoperative neurophysiologic monitoring with EMG, MEP, and SSEP was intact until the very end

Postoperative MRI showed complete resection of the tumor. The patient neurologically recovered fully after surgery.
